# Bibliometrics-guided cyberpharmacology and transcriptomics for multidimensional analysis of the antihepatic fibrosis mechanism of kaempferol

**DOI:** 10.3389/fmolb.2025.1607103

**Published:** 2025-08-29

**Authors:** Jiali Liu, Xiaowen Song, Xinni Song, Xinyue Fu, Shufang Niu, Hong Chang, Songli Shi, Meiqing Yang, Ruiqi Zhao, Peng Wang, Jun Qi, Wanfu Bai

**Affiliations:** ^1^ Department of Pharmacy, Baotou Medical College, Baotou, China; ^2^ The Second Affiliated Hospital of Baotou Medical College, Baotou, China; ^3^ The First Affiliated Hospital of Baotou Medical College, Baotou, China; ^4^ Institute of Bioactive Substance and Function of Mongolian Medicine and Chinese Materia Medica, Baotou Medical College, Baotou, China

**Keywords:** bibliometrics, network pharmacology, kaempferol, hepatic fibrosis, transcriptomics

## Abstract

**Introduction:**

Hepatic Fibrosis (HF), a pathological remodeling process triggered by persistent liver damage, is marked by the excessive buildup of extracellular matrix (ECM), leading to a gradual deterioration of liver function and an increased likelihood of advancing to cirrhosis and liver failure.

**Methods:**

This study adopts a systematic pharmacology methodology, initially employing bibliometric analysis to identify traditional Chinese medicine (TCM) formulations and individual herbs with potential anti-HF properties. Subsequently, a multi-dimensional network analysis is conducted to pinpoint core active components. Experimental investigations involve the construction of a carbon tetrachloride (CCl_4_)-induced rat model of liver fibrosis, complemented by transcriptomic technology to systematically elucidate the mechanisms of action of the active components in TCM.

**Results:**

In this study, kaempferol (KA), identified as the principal active compound with anti-fibrotic properties, was selected from traditional Chinese medicine (TCM) and TCM prescriptions through a combination of bibliometric analysis and network pharmacology. Pharmacodynamic evaluations, including pathological section analyses, demonstrated that KA effectively mitigated the fibrotic process and decreased collagen deposition. Further corroborated by ELISA experiments, kaempferol exhibited pronounced anti-fibrotic effects, inhibited inflammatory responses, restored liver function indices, and ameliorated the progression of liver fibrosis. Mechanistic investigations revealed that KA modulated fatty acid metabolism, retinol metabolism, and arachidonic acid metabolism by regulating the expression of key metabolic enzyme genes such as *SCD, SCD2, FADS2,* and *CYP4A8*, and significantly influenced the activity of the PPAR signaling pathway. Additionally, it impacted the dysregulation of lipid metabolism and inflammatory response pathways, significantly inhibited hepatic stellate cell (HSC) activation, and reduced ECM accumulation.

**Discussion:**

This finding elucidates the mechanism by which KA attenuates HF through multi-target regulation, and provides a theoretical basis for metabolic reprogramming-based therapeutic strategies with translational valu.

## Graphical Abstract

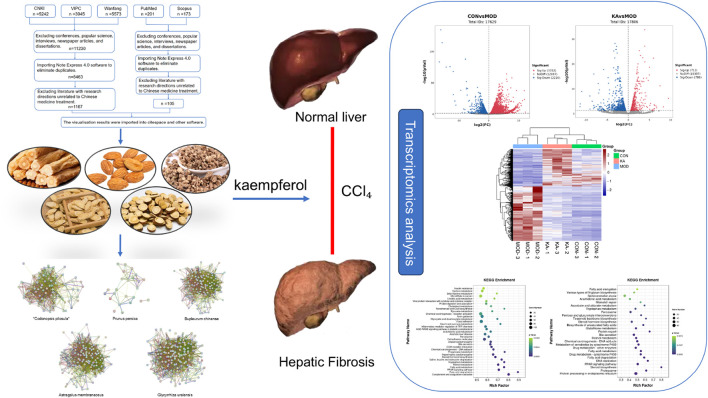

## 1 Introduction

Hepatic fibrosis (HF) is a pathological process associated with chronic liver disease, characterized by the abnormal deposition of extracellular matrix (ECM) (George et al., 2019). The pathological progression of HF encompasses inflammatory activation (Zhangdi et al., 2019), disruption of oxidative stress balance (Lin et al., 2018), and the persistent activation of pro-fibrotic signaling pathways, eventually advancing to liver failure or hepatocellular carcinoma (HCC). This condition has become a major global public health issue (Yang et al., 2023). In this context, hepatic stellate cells (HSCs) act as key effector cells, driving the fibrotic process through complex interactions with various cells, cytokines, and signaling pathways (Kitano and Bloomston, 2016). Although etiological treatment is regarded as the gold standard for preventing and managing HF (Wang et al., 2022), its effects are often slow, and it is challenging to reverse advanced fibrotic lesions. While silymarin is a widely utilized therapeutic agent for liver fibrosis, it suffers from poor oral bioavailability, rapid hepatic metabolism, and challenges in maintaining effective blood drug concentrations (Vanzan et al., 2024). The development of combination regimens with other antifibrotic agents remains in its infancy. Consequently, traditional Chinese medicine (TCM) has emerged as a significant area of interest in liver fibrosis treatment due to its multi-target regulatory properties (Jia et al., 2022).

TCM formulations facilitate systematic intervention in the pathological processes of HF through the synergistic modulation of disease-associated biological networks by multiple components (Li et al., 2022). This “multi-component, multi-pathway” mechanism of action provides unique benefits in addressing complex diseases, as it allows for a more comprehensive and synergistic therapeutic approach (Liu et al., 2024). Furthermore, TCM has been widely applied in the clinical treatment of HF due to its significant safety and economic benefits. Significant progress has been made in the research on the treatment of HF.

The effective active ingredient for the treatment of HF was identified as kaempferol (KA) through data screening of extensive literature and analysis of web-based pharmacological data. KA is an important natural flavonoid compound that is widely found in a variety of medicinal plants, including the Asteraceae family, as well as in cruciferous vegetables and rutabaga fruits (Chen et al., 2023). This compound demonstrates a wide range of biological activities, addressing critical pathological processes such as free radical scavenging, inflammation inhibition (Alam et al., 2020), and tumor cell cycle regulation (Ma et al., 2023). KA exhibits notable hepatoprotective properties by counteracting chemically induced liver damage, with its mechanism of action intricately linked to the regulation of hepatocyte metabolic homeostasis (Wang et al., 2015). In comparison to silymarin, a well-known treatment for liver fibrosis, kaempferol exhibits a broader multi-target mechanism of action, enabling a more comprehensive regulation of critical pathways involved in liver fibrosis, such as TGF-β/Smad and PI3K/Akt (El-Hawary et al., 2019; Xia et al., 2025), Kaempferol demonstrates greater potential in inhibiting the activation of hepatic stellate cells and promoting collagen degradation. Additionally, kaempferol is characterized by its wide availability and low production cost (Alam et al., 2020), making it a more economical option while maintaining efficacy. This cost-effectiveness can better accommodate the long-term medication needs of patients and enhance treatment adherence.

This study introduces an innovative research paradigm, namely, “from macro screening to micro analysis”. Initially, bibliometric techniques are employed to systematically analyze the omics characteristics of TCM anti-HF research, facilitating the identification of potential active components. Subsequently, network pharmacology is utilized to construct a multi-dimensional interaction network encompassing “component-target-pathway” (Zheng et al., 2024), relationships, thereby elucidating key pharmacodynamic substances and their central targets (Xiang et al., 2024). Concurrently, network pharmacology has demonstrated significant multi-level systematic advantages in the study of traditional Chinese medicine. Firstly, this approach allows for a comprehensive analysis of the synergistic mechanisms among multiple components and facilitates the precise optimization of compound compatibility ratios (Li et al., 2025). Secondly, it enables the efficient identification of key active ingredients within a single herb and elucidates its multi-target synergistic action mode (Su et al., 2025). Furthermore, focusing on a single compound not only broadens its potential therapeutic applications but also provides precise guidance for the optimization of its molecular structure (Zheng et al., 2025). Subsequently, *in vivo* experiments were conducted to verify the pharmacodynamic effects of KA on HF in SD rats induced by carbon tetrachloride and treated with KA, and the pharmacodynamic effects of KA on HF were analyzed by combining with pathological sections and relevant biochemical indexes, and further histological analyses were carried out to reveal the specific mechanism of KA’s antifibrotic effects. This methodological framework not only clarifies the multi-dimensional mechanisms underlying TCM but also lays a foundational theoretical groundwork for the innovation of new pharmacological treatments aimed at combating HF.

## 2 Methods

### 2.1 Bibliometric analysis

#### 2.1.1 Data sources

The literature review was conducted using data collected from five databases: CNKI, Wanfang Data, VIP, Scopus, and PubMed. The search strategy employed precise subject terms, including “hepatic fibrosis,” “liver fibrosis,” “Chinese medicine,” and “Chinese medicine prescription,” covering the period from 1 January 2018, to 1 January 2023.

#### 2.1.2 Literature screening criteria

Inclusion Criteria: (1) Clinical trials or experimental studies explicitly using TCM formulations or single herbs for HF intervention. (2) Original research with detailed prescription compositions. Exclusion Criteria: (1) Duplicate publications. (2) Studies lacking detailed prescription compositions (e.g., labeled as “standardized formula” without specific components). (3) Studies involving subjects with concurrent conditions like tumors, autoimmune diseases, or other comorbidities. (4) Non-research literature (e.g., popular science articles, interviews, conference summaries).

#### 2.1.3 Data processing

Using NoteExpress 4.0 for duplicate removal, two researchers independently reviewed titles and abstracts, with a senior researcher resolving disagreements. Documents meeting criteria were exported to Refworks format to build a structured database, and then a keyword co-occurrence network visualization was generated using VOSviewer version 1.6.19, with a minimum word frequency threshold set at five occurrences. Finally, a frequency matrix of Chinese medicine ingredients was constructed in Microsoft Excel 2021 to identify high-frequency candidates based on frequency-efficacy correlation.

### 2.2 Network pharmacology analysis

#### 2.2.1 Screening of active component and target prediction

Based on in accordance with the standards recordoutlined in the Chinese Pharmacopoeia (2020 edition), the active components of *Codonopsis pilosula*, *Prunus persica*, *Bupleurum chinense*, *Glycyrrhiza uralensis*, and *Astragalus membranaceus* were identified using the TCM Systems Pharmacology Database and Analysis Platform (TCMSP; https://tcmsp-e.com/). Following international pharmacokinetic screening criteria (Yan et al., 2018).

#### 2.2.2 Integration of disease-related targets

Disease-related targets were extracted from GeneCards (https://www.genecards.org/) V5.13 and the Online Mendelian Inheritance in Man (OMIM; https://omim.org/) database using the search terms “Hepatic Fibrosis” and “Liver Fibrosis.” The intersection of targets from TCM and disease-related targets was determined using the JVenn (Zhang Y. et al., 2022) interactive tool (https://jvenn.toulouse.inrae.fr/). Significant intersection targets were determined through hypergeometric testing, with a false discovery rate (FDR)-adjusted p -value of less than 0.05.

#### 2.2.3 Construction of multi-dimensional interaction networks

The shared targets were imported into STRING version 11.5 (https://string-db.org/), with a confidence threshold set at ≥0.4 (Martino et al., 2021), to construct a high-density protein-protein interaction (PPI) network for the organism *Homo sapiens*. Cytoscape version 3.9.1 was employed to calculate node characteristic parameters, including degree centrality, betweenness centrality, and closeness centrality. Utilizing inverse pharmacophore mapping, the top five core targets (Degree ≥2 × Median) and their corresponding binding active components were identified.

### 2.3 Experimental animals and drug preparation

This study received approval from the Experimental Animal Ethics Committee of Baotou Medical College (Approval No.: 2022-96-1). A cohort of forty healthy male Sprague-Dawley (SD) rats (*Rattus norvegicus*, strain code: VAF/Plus®), classified as SPF-grade, aged between 6 and 8 weeks and weighing 190-220 g, were procured from the National Institutes for Food and Drug Control [Laboratory Animal Production License No.: SCXK (Jing) 2017-0005]. The rats underwent a 7-day acclimatization period in a controlled environment, maintained at a temperature of 22 °C ± 1 °C and a relative humidity of 50% ± 5%, with unrestricted access to sterilized irradiated feed (AIN-93G standard formula) and drinking water with a pH of 2.5–3.0. KA, (CAS: 520-18-3, with a purity of ≥98%, sourced from Shanghai Yuanye Bio-Technology Co., Ltd.) was utilized in this study. A suspension of KA (KA701216 Shanghai Jieshikai Biotechnology Co.) at a concentration of 5 mg/mL was prepared using 0.5% sodium carboxymethyl cellulose (CMC-Na, Sigma-Aldrich, C5678) as the solvent, followed by ultrasonic oscillation at 40 kHz for 30 min. The suspension was freshly prepared prior to use and stored at 4 °C in a dark environment.

### 2.4 HF model construction and drug administration

Following a 7-day acclimatization period, the experimental animals were allocated into four groups using a stratified randomization method based on body weight: the blank control group (CON), the model group (MOD), the silymarin positive control group, and the kaempfenol intervention group (KA), with each group comprising 10 animals. The Reynaert chronic heart failure induction method, as modified, was employed (Huang et al., 2018). The MOD, silymarin positive control, and KA groups received subcutaneous injections of a 40% carbon tetrachloride (CCl_4_) suspension in corn oil (Sigma-Aldrich, 319961). An initial induction dose of 5 mL/kg was administered during the first week, followed by a maintenance dose of 3 mL/kg biweekly for a duration of 7 weeks, ensuring a minimum dosing interval of 72 h. The CON group was administered an equivalent volume of sterile corn oil. Commencing on the first day of model induction, drug interventions were conducted daily between 8:00 and 10:00 a.m. The specific intervention protocols were as follows: (1) The CON and MOD groups received 0.9% normal saline via oral gavage at a dosage of 10 mL/kg. (2) The silymarin positive control group was administered 100 mg/kg of silymarin, dissolved in a 10 mL/kg solution, via oral gavage. (3) In the kaempferol group, a suspension of kaempferol at a dosage of 100 mg/kg was dissolved in a 10 mL/kg solution and administered orally via gavage. The allocation of subjects into groups was conducted by independent researchers utilizing a computer-generated random number system. To ensure rigorous blinding, the study employed a stringent triple-blind design. Specifically, the experimental operators, responsible for drug administration and sample collection, were not involved in the grouping process and remained unaware of the treatment assignments. Histopathological evaluations were independently performed by two senior pathologists using a double-blind approach, with neither pathologist having access to the group allocation information. Data analysts were only exposed to anonymized, encoded data throughout the analysis process until the completion of statistical evaluations. The chosen dose of 100 mg/kg was selected to achieve a local hepatic concentration within the effective range of 2–10 μM, as established by *in vitro* studies. Additionally, this dosage has been demonstrated to significantly ameliorate liver fibrosis markers in several animal studies, while maintaining a favorable safety profile. This selection of dose was designed to address the requirements for anti-fibrotic therapy while ensuring the safety of medication (Cao et al., 2023) Continuous monitoring was conducted to assess the following parameters: (1) Changes in biological rhythms, including food intake and frequency of voluntary activities. (2) Evaluation of survival status, focusing on coat gloss and mucosal coloration. (3) Dynamic Body Weight Monitoring: Body weight was measured biweekly, on Mondays and Thursdays, with an accuracy of ±0.1 g.

### 2.5 Observation of liver tissue morphology and sample collection in rats

Following an 8-week regimen of continuous drug administration, rats were anesthetized 24 h after the final dose using an intraperitoneal injection of sodium pentobarbital solution (3%, 30 mg/kg). Post-euthanasia, immediate assessments of the liver’s color, texture, morphology, and volume were conducted. Subsequently, 5 mL of blood was drawn from the abdominal aorta, with serum being separated and preserved at −80 °C. A portion of the liver tissue was preserved in a 4% paraformaldehyde solution, while the remaining tissue was rapidly frozen in liquid nitrogen, transferred to cryotubes, and stored at −80 °C for subsequent analyses.

### 2.6 Determination of biochemical markers of liver fibrosis in rats

Adequate amounts of rat liver tissue from each group were taken. The levels of key biochemical indices were measured and analyzed using enzyme-linked immunosorbent assay (ELISA) with an ELISA enzyme labeled instrument, operated according to the instructions of the corresponding kit. These indices included inflammatory factors such as interleukin-1β (IL-1β) and tumor necrosis factor-α (TNF-α); liver function indices including aspartate aminotransferase (AST) and alanine aminotransferase (ALT); and fibrosis-related indices such as hyaluronic acid (HA), laminin (LN), type III procollagen (PC-III), and type IV collagen (COL-IV) in the liver tissues.

#### 2.6.1 Sample preparation and sequencing

Upon completion of the experiment, three biological replicate samples were collected from each experimental group (CON, MOD, KA) and stored at −80 °C for no longer than 6 weeks. Total RNA was extracted utilizing the TRIzol® method, and any genomic DNA contaminants were removed using DNase I (RNase-free, Thermo Fisher, EN0521). RNA integrity was evaluated via 1% agarose gel electrophoresis (100 V, 30 min), ensuring a 28S/18S ratio of ≥1.8, while RNA purity was assessed using the NanoDrop 2000 spectrophotometer (Thermo Fisher), with acceptable A260/A280 ratios ranging from 1.8 to 2.1 and A260/A230 ratios of ≥2.0. The RNA Integrity Number (RIN) was assessed utilizing the Agilent 2100 Bioanalyzer with the RNA Nano 6000 chip, where RIN values of 7.0 or higher were deemed acceptable. mRNA sequencing libraries were prepared in accordance with the standard protocol of the NEBNext® Ultra^TM^ II RNA Library Prep Kit (NEB, E7770S). Sequencing was executed on the Illumina NovaSeq 6000 platform, employing 150 base pair paired-end reads (2 × 150 PE).

#### 2.6.2 Bioinformatics analysis

In this study, we employed FASTP (version 0.19.7) for the initial processing of raw sequencing data, specifically the raw reads. During this phase, sequences of low quality (phred score <20) and contaminating adapter sequences were excised, resulting in a dataset of cleaned reads with a clean data ratio of ≥95%. Subsequently, these cleaned reads were aligned to the reference genome, *Rattus norvegicus* mRatBN7.2, utilizing HISAT2 (version 2.2.1). The calculation of Fragments Per Kilobase of transcript per Million mapped reads (FPKM) values was conducted using StringTie (version 2.1.7). For datasets comprising biological replicates, differentially expressed genes (DEGs) were identified using EdgeR (version 3.42.4), applying thresholds of a false discovery rate (FDR) < 0.05 and an absolute log2 fold change (|log2FC|) ≥ 1. Functional enrichment analyses of Gene Ontology (GO) terms and Kyoto Encyclopedia of Genes and Genomes (KEGG) pathways were conducted utilizing ClusterProfiler (version 4.6.2), with an adjusted p-value threshold of <0.05. Visualization of the regulatory and clustering characteristics of differentially expressed mRNAs was achieved through the generation of volcano plots and correlation heatmaps using the OmicStudio cloud platform (https://www.omicstudio.cn/tool).

### 2.7 Statistical analysis

Statistical evaluations were performed utilizing SPSS software, version 26.0. Experimental data were presented as mean ± standard deviation ( $\bar{x}$ ± s). Comparisons among multiple groups were performed using one-way analysis of variance (ANOVA), with the assumption of homogeneity of variance verified by Levene’s test. For cases where variance was homogeneous, the Least Significant Difference (LSD) method was employed for pairwise comparisons; in instances of heterogeneous variance, Dunnett’s T3 method was utilized for multiple comparisons. Pairwise comparisons within groups were conducted using the non-parametric Wilcoxon rank-sum test. All statistical analyses employed a two-sided approach, establishing a significance level at α = 0.05. A P-value below 0.05 was deemed representative of statistical significance.

## 3 Results

### 3.1 Fundamental characteristics of the literature on TCM in the treatment of HF

**FIGURE 1:**
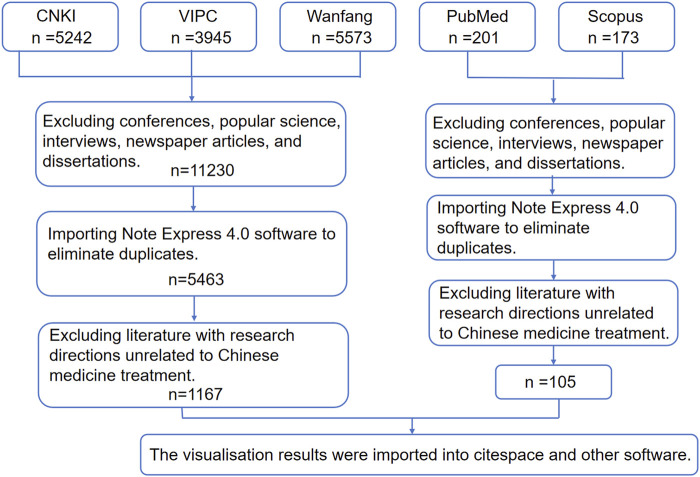
The literature screening process.

This study initially identified a total of 14,760 publications related to the application of TCM in the treatment of HF through a systematic search of domestic databases. The literature was subsequently filtered based on predefined inclusion and exclusion criteria. Initially, 3,530 non-research documents, including conference abstracts, popular science articles, news reports, and dissertations, were excluded. Following this, 5,767 duplicate entries were removed using the Note Express 4.0 literature management software. Additionally, 4,296 articles that did not pertain to TCM treatment of HF were excluded. In PubMed, the search formula “(TCM) AND (liver fibrosis) AND (hepatic fibrosis)” yielded 134 articles, whereas the formula “((TCM prescription) AND (liver fibrosis)) AND (liver fibrosis)” retrieved 37 articles. In Scopus, the corresponding search methods resulted in 102 and 71 articles, respectively. After excluding incomplete, irrelevant, and duplicate articles, the total number of relevant articles was reduced to 105, which constitutes only 8% of similar studies found in the Chinese databases. Consequently, our study undertook a comprehensive analysis of the literature available in CNKI, Wanfang, and VIP, while also incorporating relevant international studies from databases such as PubMed and Scopus. Ultimately, 1,167 papers that satisfied the criteria were retained for analysis. Figure 1 illustrates the detailed procedure involved in the literature screening process.

### 3.2 Keyword analysis

#### 3.2.1 Keyword Co-occurrence analysis

**FIGURE 2:**
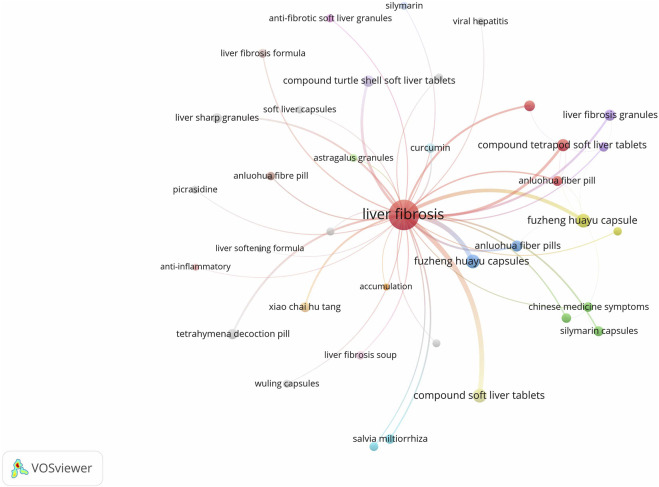
Keyword co-occurrence mapping.

Keywords were analyzed for co-occurrence using VOSviewer 1.6.19 software, with the frequency of keywords serving as an indicator of research interest in the field. The database included a total of 1,816 keywords, of which 153 appeared with a frequency of five or more. A co-occurrence analysis was conducted on keywords associated with herbal prescriptions and monomers, resulting in the generation of a keyword co-occurrence network graph (Figure 2). In this graph, each node represents a keyword, and the size of the node corresponds to the frequency of the keyword. The connecting lines between nodes signify the co-occurrence relationships between the keywords. Notably, keywords such as “Compound Jianjia Soft Liver Tablets”, “Fuzheng Huayu Capsule”, “Tetrapod Decoction Pill”, “Anluohua Fibre Pill”, “Fuzheng Huayu Capsules”, “Baojia Decoction Pills”, “Anluo Huayu Pills”, and “Huangqi” exhibit higher frequencies of occurrence. Statistical analysis of keyword frequencies reveals the top 25 single drugs, as presented in Table 1.

**TABLE 1:** Ranking of single flavour drugs (top 25).

Traditional Chinese medicine	Frequency
*Codonopsis pilosula*	273
*Prunus persica*	170
*Bupleurum chinense*	168
*Astragalus membranaceus*	135
*Glycyrrhiza uralensis*	135
*Radix Salviae*	132
*Angelicae Sinensis Radix*	131
*Panax Notoginseng (Burk.) F. H. Chen Ex C. Chow*	122
*Paeoniae Radix Alba*	121
*Radix Rhei Et Rhizome*	113
*Carapax Trionycis*	111
*Radix Paeoniae Rubra*	108
*Atractylodes Macrocephala Koidz.*	86
*Schisandrae Chinensis Fructus*	83
*Cortex Moutan*	83
*Isatidis Radix*	79
*Gynostemmae Pentaphylli Herba*	77
*Scutellariae Radix*	76
*Curcumae Radix*	73
*Placenta Hominis*	68
*Forsythiae Fructus*	68
*Corayceps*	68
*Pine Pollen*	66
*Rehmanniae Radix Praeparata*	64
*Arum Ternatum Thunb.*	61

### 3.3 Acquisition of active ingredients and targets

A total of 92 active ingredients were identified from *Glycyrrhiza uralensis*. After integrating the active ingredient targets and eliminating duplicate entries, 183 unique targets were obtained. In the study of *Codonopsis pilosula*, 21 active ingredients were identified. Upon integrating the active ingredient targets and eliminating duplicates, 85 unique targets were obtained. *Astragalus membranaceus* was found to contain 20 active ingredients, resulting in 163 unique targets after target integration and duplicate removal. The investigation of *Prunus persica* revealed 23 active ingredients, leading to 31 unique targets after processing. Conversely, *Bupleurum chinense* comprised 17 active ingredients, yielding 150 unique targets following the same procedure.

### 3.4 Common targets of five TCMs with HF

**FIGURE 3:**
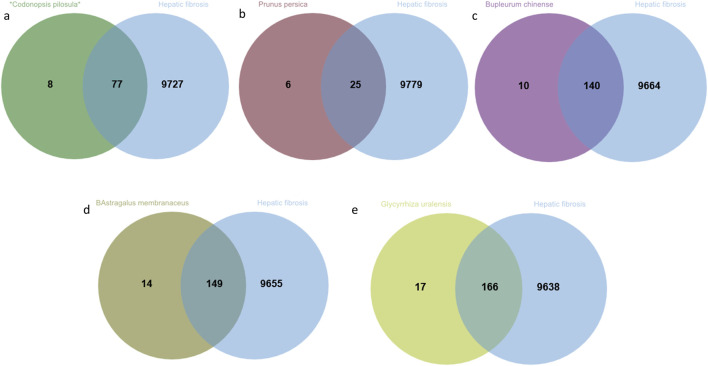
The intersecting targets of *Codonopsis pilosula*、*Prunus persica*、*Bupleurum chinense*、*Astragalus membranaceus* and *Glycyrrhiza uralensis* with HF. **(a)** *Codonopsis pilosula* and HF; **(b)** *Prunus persica* and HF; **(c)** *Bupleurum chinense* and HF; **(d)** *Astragalus membranaceus* and HF; **(e)** *Glycyrrhiza uralensis* and HF.

In this study, a total of 9,804 targets associated with HF were identified. Common targets were determined using Venny 2.1 to find intersections with each TCM (Figure 3, Supplementary Material 1).

### 3.5 Protein-protein interaction (PPI) network of five Chinese herbal medicines for the treatment of HF

**FIGURE 4:**
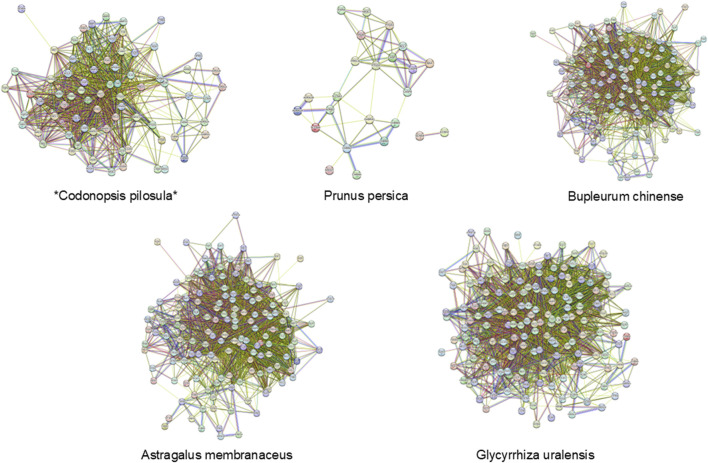
The protein interaction network of *Codonopsis pilosula*, *Prunus persica*, *Bupleurum chinense*, *Astragalus membranaceus* and *Glycyrrhiza uralensis* in the treatment of HF.

After uploading the intersecting targets identified from the screening to the STRING database (https://string-db.org/), with the species specified as “*Homo sapiens*” and hidden no-joints, a protein-protein interaction (PPI) network diagram was generated (Figure 4). Through analysis of the interaction scores between the nodes, the top five core targets were identified, including 25 key targets such as GAPDH, TP53, Akt1, IL-6, and TNF. Subsequently, the active ingredients of TCMs associated with these targets were further screened, and their frequency of occurrence was quantified (Table 2). Notably, compounds such as quercetin, KA, and β-sitosterol, found in TCMs like *Codonopsis pilosula*, *Prunus persica*, *Bupleurum chinense*, *Astragalus membranaceus* and *Glycyrrhiza uralensis* exhibited high target binding frequency and demonstrated significant correlation within the target network.

**TABLE 2:** Target active ingredients and frequency.

Traditional Chinese medicine	Node 1	Node 2	degree	Chemical compound	
*Codonopsis pilosula*	TP53	MDM2	0.99	luteolin	luteolin
*Codonopsis pilosula*	TP53	BCL2L1	0.99	luteolin	luteolin
*Codonopsis pilosula*	TP53	CDKN1A	0.99	luteolin	luteolin
*Codonopsis pilosula*	RXRA	PPARG	0.99	Perlolyrine	luteolin
*Prunus persica*	CASP9	CASP3	0.99	beta-sitosterol	beta-sitosterol
*Prunus persica*	CASP8	CASP3	0.99	beta-sitosterol	beta-sitosterol
*Prunus persica*	BCL2	BAX	0.99	beta-sitosterol	beta-sitosterol
*Bupleurum chinense*	VCAM1	ICAM1	0.99	kaempferol	kaempferol
*Bupleurum chinense*	TP53	HIF1A	0.99	quercetin	quercetin
*Bupleurum chinense*	TP53	BCL2	0.99	quercetin	kaempferol
*Astragalus membranaceus*	VCAM1	ICAM1	0.99	kaempferol	kaempferol
*Astragalus membranaceus*	TP53	HIF1A	0.99	quercetin	quercetin
*Astragalus membranaceus*	TP53	BCL2	0.99	quercetin	kaempferol
*Glycyrrhiza uralensis*	VCAM1	ICAM1	0.99	kaempferol	kaempferol
*Glycyrrhiza uralensis*	TP53	HIF1A	0.99	quercetin	quercetin
*Glycyrrhiza uralensis*	TP53	BCL2	0.99	quercetin	kaempferol

### 3.6 Morphological alterations in rat liver tissue appearance

**FIGURE 5:**
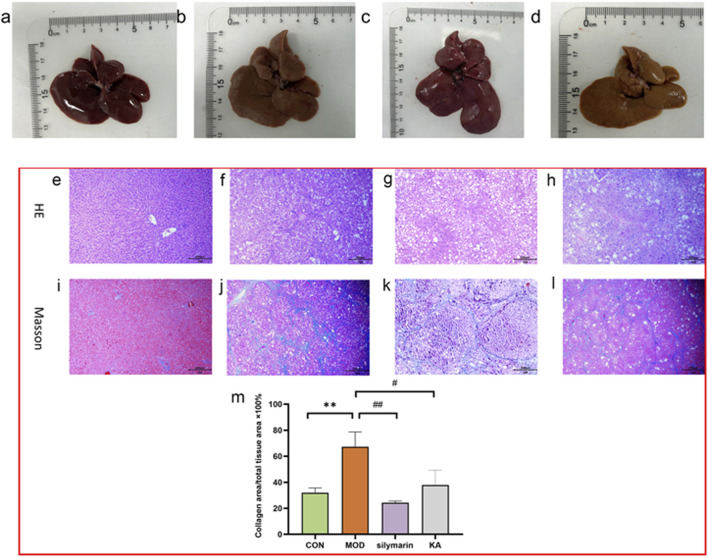
Histopathological evaluation of rat liver tissue. **(a–d)** Macroscopic appearance and morphology of liver tissue: **(a)** Normal group, **(b)** Model group, **(c)** Silymarin-treated group, **(d)** KA-treated group. **(e–l)** Histopathologic analysis by H&E and Masson trichrome staining: **(e,i)** The normal group; **(f,j)** The model group; **(g,k)** The silymarin group; **(h,i)** The KA treatment group. **(m)** Histopathological scores among each group. Comparisons with the normal control group, the statistical significance indicated by ***P < 0.001, **P < 0.01, and *P < 0.05. Comparisons with the model group are indicated by ###*P* < 0.001, ##*P* < 0.01, and #*P* < 0.05.

In the CON group, the liver tissue of normal rats exhibited a reddish-brown color, uniformity, a smooth surface, and a soft texture, as depicted in Figure 5a. Conversely, the livers of rats in the MOD group presented a brownish-yellow hue and were swollen, with an uneven, fibrotic surface and a hard texture, characterized by blotches and an inconsistent color, as shown in Figure 5b. Notably, there was a significant improvement in liver color and texture in the silymarin group (Figure 5c) as well as in the KA group (Figure 5d).

### 3.7 Histological changes in rat liver

Histological examination of normal rat liver revealed structurally intact tissue. However, administration of CCl_4_ for 8 weeks in the model group led to extensive HF, as determined by both qualitative and quantitative histopathological assessments. In comparison to the normal liver morphology (Figures 5e, i), CCl_4_-induced fibrosis was characterized by disrupted tissue architecture, fiber extension, formation of large fibrous septa, separation into pseudolobules, and collagen accumulation (Figures 5f,j). In the liver slices of rats that were treated with silymarin (Figures 5g,k), KA (Figures 5h,i), and CCl_4_ respectively for 8 weeks, these changes were significantly reduced. The scores for the KA-treated group demonstrated a significant improvement (*P* < 0.05) compared to the fibrotic group, as assessed by the Noetherian index of HF. However, statistically significant differences remained between the MOD and CON groups and between the MOD and silymarin groups, while no differences were detected between the scores of the CON and KA groups (Figure 5m).

### 3.8 Determination of biochemical markers of liver fibrosis in rats

**FIGURE 6:**
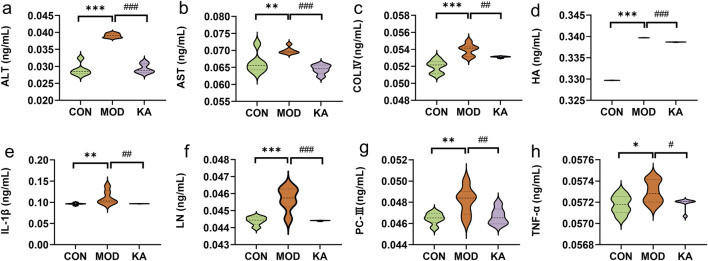
Analysis of biochemical markers. **(a)** ALT; **(b)** AST; **(c)** COL-Ⅳ; **(d)** HA; **(e)** IL-1β; **(f)** LN; **(g)** PC-Ⅲ; **(h)** TNF-α. Statistical comparison with the normal control group is denoted as follows: ***P < 0.001; **P < 0.01; *P < 0.05. Comparisons with the model group are indicated as ###*P* < 0.001; ##*P* < 0.01; #*P* < 0.05. CON: the control group, MOD: the model group, and KA: the kaempferol-administration group.

Relative to the CON group, the serum levels of ALT (0.03907 ± 0.00062 ng·mL^−1^), HA (0.33969 ± 0.00001 ng·mL^−1^), LN (0.04569 ± 0.00069 ng·mL^−1^) and COL-IV (0.05395 ± 0.00069 ng·mL^−1^) were significantly elevated (*P* < 0.001) in the model group. Additionally, the levels of IL-1β, AST and PC-III (*P* < 0.01), and TNF-α (*P* < 0.05) were also significantly increased. Conversely, the serum concentrations of ALT (0.02930 ± 0.00134 ng·mL^−1^), AST (0.06445 ± 0.00134 ng·mL^−1^), HA (0.33867 ± 0.00003 ng·mL^−1^), LN (0.04441 ± 0.00002 ng·mL^−1^) (*P* < 0.001), COL-IV, PC-III, IL-1β (*P* < 0.01), and TNF-α (*P* < 0.05) were significantly reduced in the KA group compared to the MOD group (Figure 6).

### 3.9 Transcriptomic analysis of KA-Treated HF rats

**FIGURE 7:**
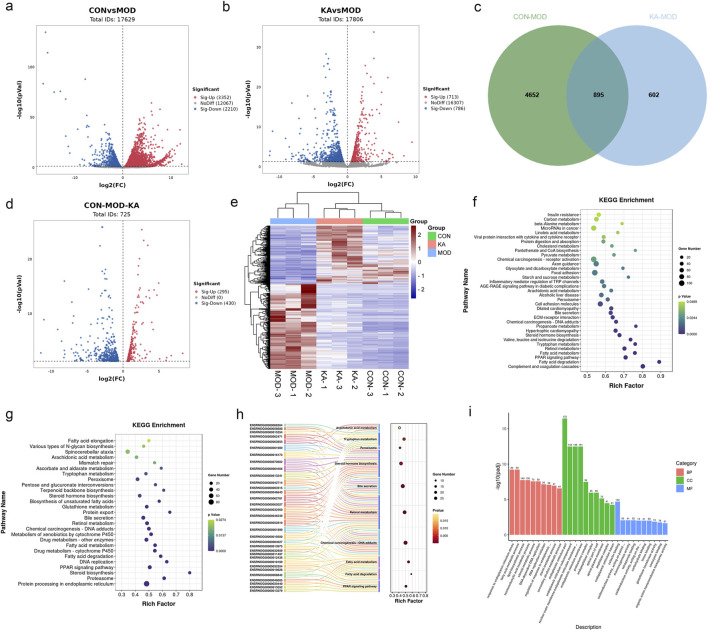
The transcriptomic analysis results of kaempferol - treated HF rats. **(a)** Volcano plot of DEGs between the CON and MOD groups; **(b)** Volcano plot of DEGs between the KA and MOD groups; **(c)** The intersection of DEGs among the CON, MOD, and KA groups; **(d)** The regulatory changes of DEGs between the CON and the MOD group after KA administration; **(e)** Clustering heatmap to analyze the regulation of differentially expressed gene content among the CON, MOD, and KA groups, with red indicating high expression and blue indicating low expression; **(f)** KEGG enrichment analysis of DEGs between the CON and MOD groups; panel; **(g)** KEGG enrichment analysis of DEGs between the KA and MOD groups; **(h)** Sankey bubble map of the intersection enrichment pathway among the CON, MOD, and KA groups; and panel; **(i)** GO functional analysis of DEGs. CON: the control group, MOD: the model group, and KA: the KA-administration group.

The study involved the analysis of the control (CON), model (MOD), and KA-treated groups to identify DEGs. The volcano plot depicted in Figure 7a illustrates that 5,562 DEGs were significantly expressed between the control and model groups, comprising 3,352 upregulated and 2,210 downregulated genes. Similarly, the volcano plot in Figure 7b reveals that 1,499 DEGs were significantly expressed between the model and KA-treated groups, with 713 genes upregulated and 786 downregulated. A total of 895 DEGs were identified across the CON, MOD, and KA groups, as illustrated by the Venn diagram in Figure 7c. Following KA treatment, the expression of 725 DEGs was restored, as shown in Figure 7d, with 295 genes upregulated and 430 downregulated (Figure 7e). These findings suggest that KA treatment may normalize certain gene expression disorders. To further elucidate the potential mechanisms underlying changes in DEGs, all prospective biomarkers underwent KEGG enrichment and functional analyses. This process identified several major enriched pathways at the transcriptomic level in both the CON and MOD groups (Figure 7f), including tryptophan metabolism, complement and coagulation cascades, fatty acid degradation, the PPAR signaling pathway, and fatty acid metabolism, among others. Similarly, the major enriched pathways identified at the transcriptomic level in the KA and MOD groups (Figure 7g) encompassed steroid biosynthesis, protein processing in the endoplasmic reticulum, the PPAR signaling pathway, and protein export, among others. Notably, ten enriched pathways were identified, including arachidonic acid metabolism, tryptophan metabolism, steroid hormone biosynthesis, retinoid metabolism, fatty acid metabolism, fatty acid degradation, and the PPAR signaling pathway. The ten enrichment pathways identified exhibited significant overlap with the primary enrichment pathways observed in the CON and MOD groups. These pathways were subsequently analyzed to elucidate the roles of the *CYP4A8*, *SCD*, *FADS2*, and *SCD2* genes in processes such as fatty acid degradation, the PPAR signaling pathway, fatty acid metabolism, retinol metabolism, and arachidonic acid and tryptophan metabolism. Additionally, retinol metabolism, arachidonic acid metabolism, and other enrichment pathways (as depicted in Figure 7h) demonstrated enhanced relevance within the transglutomics mechanism associated with KA treatment for liver fibrosis. Further investigation into the gene functions was conducted using Gene Ontology (GO) analysis on the DEGs from the MOD and KA groups. The GO enrichment analysis (illustrated in Figure 7i) revealed that the enriched biological functions predominantly involved responses to endoplasmic reticulum stress, fatty acid metabolic processes, components of the endoplasmic reticulum, binding to cofactors, oxidoreductase activity acting on paired donors, and interactions with or reduction of molecular oxygen.

### 3.10 Regulation of gene expression by KA in HF rats

**FIGURE 8:**
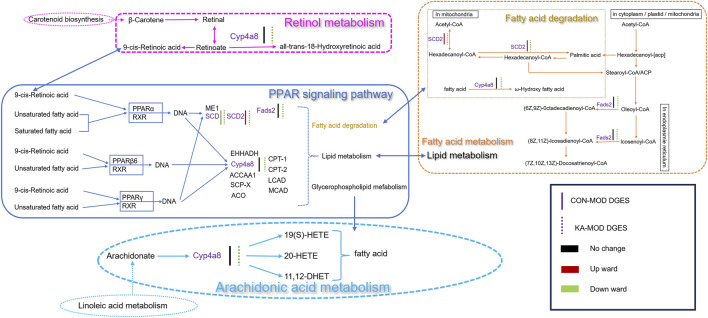
The relevance analysis mechanism diagram. CON: the control group, MOD: the model group, and KA: the kaempferol-administration group.

Through comprehensive analyses, this study identified ten key signaling pathways that are closely associated with the CCl_4_-induced HF rat model. In this study, particular attention was directed towards the DEGs within these pathways, with an emphasis on the signaling pathways significantly implicated in the pathological processes of HF, as identified through KEGG database analysis. Four DEGs were identified as playing critical regulatory roles in the pathways of fatty acid degradation, PPAR signaling, fatty acid metabolism, retinol metabolism, and arachidonic acid metabolism. Notably, Stearoyl-CoA Desaturase 2 (*SCD2*) exhibited a marked upregulation in expression within the model group, whereas its expression was significantly downregulated following treatment with the intervention drug, KA. Furthermore, Cytochrome P450 Family 4 Subfamily A Member 8 (*CYP4A8*) and Fatty Acid Desaturase 2 (*FADS2*) did not demonstrate significant changes in expression within the model group; however, their expression levels were significantly diminished post-kaempferol intervention. Concurrently, the expression of the *SCD* gene was downregulated in the model group and further decreased following kaempferol treatment (Figure 8).

## 4 Discussion

This research investigates the pathological mechanisms underlying HF and explores intervention strategies rooted in TCM. HF, a common pathological response to chronic liver injury, involves the excessive accumulation of ECM resulting from metabolic dysregulation. The excessive accumulation of type I collagen (collagen I) serves as the primary molecular foundation for liver tissue remodeling (Kisseleva, 2016). This pathological process is predominantly driven by persistent factors such as viral hepatitis and alcoholic liver injury, and its global prevalence has markedly increased in conjunction with the rising incidence of metabolic syndrome (Altamirano-Barrera et al., 2019). At the cellular and molecular levels, the phenotypic transformation of HSCs plays a central role in the development and progression of fibrosis. When exposed to a chronic inflammatory microenvironment, HSCs transition from vitamin A-rich quiescent cells into myofibroblasts, acquiring proliferative, migratory, and contractile characteristics. This transformation promotes an imbalance in ECM synthesis and degradation through signaling pathways such as TGF-β/Smad (VanHook, 2019). TCM offers distinct theoretical advantages and extensive practical experience in the prevention and treatment of HF. It operates under guiding principles such as “promoting blood circulation to remove stasis” and “soothing the liver to regulate qi.” TCM formulations, including Biejiajian Pills and Fuzheng Huayu Formula, as well as monomeric components like tanshinone IIA and curcumin, have been clinically validated to significantly enhance liver histopathological scores and serum fibrosis markers (Zhang and Schuppan, 2014; Liu et al., 2018). This study utilizes a systems pharmacology approach, integrating molecular docking and network analysis, to elucidate the dynamic network mechanisms through which TCM active components modulate HSC activation and ECM metabolism via multi-target synergistic effects.

Additionally, we utilize integrated bibliometric and network pharmacology approaches to systematically explore the current research landscape and mechanistic foundations of TCM in the management of HF. Initially, a bibliometric analysis was conducted, integrating keyword co-occurrence and timeline mapping of dynamic changes, to utilize visualization techniques for a comprehensive elucidation of research hotspots and developmental trends concerning TCM formulations and individual herbs in the treatment of HF. Subsequently, network pharmacology methods were employed to identify TCM components with potential anti-hepatic fibrosis activity, which were then experimentally validated using a rat model of HF induced by a combination of corn oil and CCl_4_. Additionally, transcriptomic analysis was utilized to thoroughly investigate the molecular mechanisms and associated gene targets through which these active TCM components modulate HF. The results demonstrate that active components of TCM exhibit anti-hepatic fibrosis effects through multi-target and multi-pathway mechanisms. The experimental findings of this study not only offer scientific validation for understanding the pharmacological mechanisms of TCM active components in treating HF but also lay a critical theoretical groundwork for further in-depth research and clinical applications of TCM in this domain.

To enhance the recognition of TCM in the treatment of HF and to ensure the adequacy and credibility of references, this study performed a comprehensive search across three authoritative databases: CNKI, the Wanfang Data Knowledge Service Platform, and the VIP Network. This comprehensive literature review facilitated the identification of research hotspots related to TCM formulations and their active components through keyword visualization analysis. The study identified five high-frequency TCM components: *Codonopsis Radix*, *Persicae Semen*, *Radix Bupleuri*, *Hedysarum Multijugum Maxim.*, and *Glycyrrhiza uralensis Fisch*. Utilizing network pharmacology approaches, a protein-protein interaction (PPI) network was developed, allowing for the identification of primary TCM components with potential anti-hepatic fibrosis activity based on relevant scoring systems. In our study, we concentrated on compounds that have been thoroughly documented in the existing literature for their specific therapeutic effects pertinent to our research objectives. Although quercetin and β-sitosterol are indeed noteworthy, our preliminary analyses revealed that the compounds we selected demonstrated a more direct relevance to the mechanisms we intended to investigate. Among these components, KA emerged as the most frequently occurring. KA, a flavonoid secondary metabolite, is extensively distributed across various plant species (Chen et al., 2023). Previous studies have highlighted its notable biological activities, such as anti-inflammatory, antioxidant, and anticancer properties (Periferakis et al., 2023). In recent years, significant attention has been directed towards the pharmacokinetic properties of kaempferol (KA) and its potential therapeutic applications in liver disease treatment. Through a systematic investigation of the oral pharmacokinetics and tissue distribution of KA and its metabolites, Liu Y. et al. (2022) identified a distinctive triple cycle pathway for this compound, which markedly enhances the local bioavailability of flavonoids in target tissues and extends their retention time in the liver and intestine (Liu X. et al., 2022). Complementary findings by Barve et al. (2009) demonstrated that kaempferol achieves peak plasma concentration within 1–2 h post-oral administration, indicative of its rapid absorption characteristics (Barve et al., 2009). Nevertheless, kaempferol undergoes extensive metabolic transformations, such as glucuronidation in the gastrointestinal tract and liver, which result in its relatively low systemic bioavailability. In a 2016 study utilizing a rat model, Zhou, Wang, and colleagues demonstrated that the oral bioavailability of kaempferol (KA) ranged from only 2.1%–3.1%. Despite this low bioavailability, subsequent toxicological assessments confirmed a favorable safety profile for KA, as no significant adverse effects on liver and kidney function were observed (Zhou et al., 2016). More recently, a 2023 study by Kim, Song, and collaborators revealed that kaempferol effectively mitigated hepatocyte necrosis and inhibited inflammatory responses in a carbon tetrachloride (CCl_4_)-induced liver injury model, thereby exhibiting significant hepatoprotective properties (Kim et al., 2023). Collectively, these findings indicate that kaempferol’s distinctive pharmacokinetic characteristics, coupled with its pronounced liver-protective efficacy, render it a highly promising candidate for the treatment of hepatic fibrosis (HF), notwithstanding its limited systemic bioavailability. To substantiate the anti- Hepatic fibrosis effects of KA, this study utilized a CCl_4_-induced rat model of HF. Through transcriptomic analysis, the study elucidated the molecular mechanisms by which KA modulates key genes and associated signaling pathways involved in HF. These findings provide critical experimental evidence supporting its potential clinical application.

The experiment revealed the pharmacodynamic changes of KA in anti-liver fibrosis. Clinically, ALT and AST are vital indicators of liver function. ALT is predominantly found in the cytoplasm of hepatocytes, with intracellular concentrations that are 1,000–3,000 times higher than those in serum (George et al., 2022). A mere 1% damage to hepatocytes can result in a doubling of serum enzyme levels. AST is most prevalent in myocardial cells, followed by the liver, skeletal muscle, and kidneys. Within hepatocytes, AST is predominantly located in the mitochondria, with a smaller fraction present in the cytoplasm. In cases of severe hepatocyte damage, the integrity of mitochondrial membranes is compromised, leading to the release of AST into the bloodstream and a consequent elevation in serum AST activity (Lavrynenko et al., 2020). IL-1β and TNF-α are critical inflammatory cytokines (Kaffe et al., 2018), that exhibit a positive correlation with inflammatory responses. Markers of serum fibrosis include HA, LN, PC-III, and COL-IV. PC-III, COL-IV, and LN constitute major structural elements of the basement membrane, playing roles in cell adhesion and signal transduction. Their overexpression during pulmonary fibrosis results in aberrant repair of the basement membrane (Ohta et al., 1995; Florea et al., 2016), thereby exacerbating the progression of fibrosis. Hyaluronic acid (HA), a polysaccharide abundantly found in the ECM, accumulates during inflammation and fibrosis, potentially contributing to tissue repair and fibrotic processes (Yang et al., 2019). The experimental findings indicated that serum biochemical markers were markedly elevated in the MOD group but decreased to levels comparable to the CON group following KA administration. Histological evaluations using H&E and Masson staining revealed substantial damage to the alveolar interstitium and liver tissue architecture in the MOD group, in contrast to normal liver tissue. Post-KA administration, the damage was significantly ameliorated, and liver tissue morphology was restored to a condition resembling that of the CON group. These results collectively confirm the successful establishment of a CCl_4_-induced rat model of liver fibrosis and demonstrate that KA exerts a notable ameliorative and therapeutic effect on liver fibrosis.

Transcriptome analysis of KA against HF in the present study showed that the regulation of KA affected the relevant metabolic and signaling pathways. The degradation and metabolism of aliphatic acids are critically involved in the initiation and progression of HF. As the primary metabolic organ, the liver possesses the ability to synthesize, decompose, and metabolize aliphatic acids (Heeren and Scheja, 2021). Under pathological conditions such as obesity, hyperlipidemia, and, the liver often exhibits excessive lipid accumulation (Heeren and Scheja, 2021). The expansion of adipocytes, infiltration of leukocytes, and release of cytokines collectively contribute to lipid accumulation in hepatocytes, thereby exacerbating the onset and progression of HF (Shen et al., 2019). Furthermore, disruptions in fatty acid degradation and metabolism can induce oxidative stress and inflammatory responses, further accelerating the development of HF (Li et al., 2024). This study identifies *SCD2*, *FADS2*, and the PPAR signaling pathway as pivotal regulators of aliphatic acid synthesis and metabolism. *SCD2*, a member of the stearoyl-CoA desaturase family serves a vital function as the rate-limiting enzyme in the synthesis of monounsaturated fatty acids (MUFAs) (O’Neill et al., 2020), thereby promoting aliphatic acid synthesis and participating in various metabolic processes within hepatocytes. Its expression level is positively correlated with the severity of HF. *FADS2* is a crucial enzyme involved in the desaturation of aliphatic acids and is essential for the biosynthesis of long-chain polyunsaturated fatty acids (PUFAs) (Yamane et al., 2021). The upregulation of *FADS2* expression markedly promotes the progression of HF, resulting in the accelerated synthesis of unsaturated fatty acids and a disruption of intracellular lipid metabolism within hepatocytes (Kothapalli et al., 2023). The PPAR signaling pathway constitutes the core regulatory network of fatty acid metabolism, and its activation can exert anti-liver fibrosis effects through a multi-target synergistic mechanism. Research has demonstrated that KA significantly alters hepatic lipid metabolism by down-regulating the expression of the SCD and CYP4A8 genes. The inhibition of the SCD gene leads to a reduction in the production of monounsaturated fatty acids and diminishes the activation of HSC through lipotoxicity (Subedi et al., 2020). KA suppresses the expression of its downstream target, Cyp4a, through the downregulation of SCD2 and the activation of the PPARα signaling pathway. This process results in a reduction of ROS production, alleviation of oxidative stress-induced hepatocyte injury, and a consequent decrease in serum ALT/AST levels (Shearn et al., 2014). Concurrently, the downregulation of CYP4A8 decreases the excessive production of ω-hydroxylated fatty acids (Gato et al., 2012). Collectively, these changes alleviate endoplasmic reticulum stress and oxidative damage caused by abnormal lipid accumulation. This metabolic remodeling further activates the PPARα/γ isoforms: activation of PPARα enhances fatty acid β-oxidation, accelerates lipid clearance, and reduces lipid peroxide accumulation (Zhang Z. et al., 2022). Simultaneously, PPARγ nuclear translocation not only improves insulin sensitivity but also inhibits HSC activation and collagen deposition by directly suppressing the TGF-β/Smad pathway. Furthermore, the synergistic activation of PPARβ/δ can inhibit inflammatory pathways, such as NF-κB, and reduce the release of pro-fibrotic factors, including TNF-α and IL-6. This establishes a cascade reaction network characterized by “metabolic regulation, inflammation suppression, and fibrosis remission.” (Titus et al., 2023). This study investigates the complex regulatory mechanisms that confer anti-liver fibrosis effects by enhancing liver lipid homeostasis, mitigating oxidative stress, and inhibiting HSC activation. KA mitigates ECM deposition through a multi-target regulatory mechanism by inhibiting *SCD2*, which is crucial for lipid metabolic reprogramming in HSC activation. This inhibition prevents the lipid-dependent transformation of HSCs into myofibroblasts and reduces ECM component secretion like COL-IV and LN. Concurrently, *FADS2* and *CYP4A8* facilitate HSC proliferation and fibrosis via the TGF-β1 and MAPK/ERK pathways, respectively. KA effectively inhibits both *FADS2* and *CYP4A8*, blocking these signaling pathways, thereby reducing HSC activation and fibrosis markers such as HA and PC-III, demonstrating its anti-liver fibrosis properties (Yan et al., 2022; Li et al., 2020).

Furthermore, retinol metabolism and arachidonic acid metabolism are integral to the potential therapeutic effects of KA on HF. Research has demonstrated that retinol metabolism is integral to liver regeneration and the mechanisms underlying disease development, encompassing processes such as inflammation, fatty liver disease, fibrosis, and cirrhosis (Wang et al., 2023). Exposure of the liver to CCl_4_ results in hepatocyte damage and the activation of hematopoietic stem cells, concurrently disrupting retinol metabolism. These alterations further exacerbate the progression of HF and cirrhosis (Ping et al., 2023). Enzymes and bioactive products derived from arachidonic acid metabolism are indicative of inflammatory responses (Liu X. et al., 2022). *CYP4A8*, a cytochrome P450 enzyme from the *CYP4A* subfamily, is primarily expressed in the liver and plays a role in fatty acid metabolism, particularly in the ω- and (ω-1)-hydroxylation of long-chain fatty acids (Yamaguchi et al., 2002). CYP4A8 may possess anti-inflammatory properties by modulating the metabolism of fatty acids, arachidonic acid, and retinoic acid, thereby playing a pivotal role in mitigating HF (Nakamura et al., 1994; Hoch et al., 2000).

This study utilized transcriptomic sequencing technology in conjunction with experimental validation methods to systematically examine the therapeutic effects of KA on CCl_4_-induced HF in rats, as well as its underlying pharmacological mechanisms. The findings indicated that the therapeutic efficacy of KA is intricately linked to the regulation of aliphatic acid degradation and metabolism, retinol metabolism, and arachidonic acid metabolism. KA significantly downregulated the expression of key genes such as *SCD*, *SCD2*, *FADS2*, and *Cyp4A8*, inhibited the release of inflammatory cytokines in hepatic tissue, and reduced the synthesis of aliphatic acids in the liver. These effects collectively helped reduce oxidative stress and inflammatory responses, thereby promoting the recovery of CCl_4_-induced liver damage. Additionally, KA may further impact lipid metabolism and inflammatory responses through modulation of the PPAR signaling pathway, offering a molecular mechanism-based rationale for its potential application in the treatment of HF. This research elucidates the potential mechanisms by which KA may contribute to the treatment of HF, while also identifying significant molecular targets and providing theoretical foundations for the development of innovative anti-fibrotic therapeutic strategies. CCl_4_ is a well-established hepatotoxic compound frequently utilized in experimental models to induce liver fibrosis, thereby facilitating the study of chronic liver disease pathogenesis. Nonetheless, it is crucial to recognize that CCl4 can also induce pulmonary fibrosis, a phenomenon that is not yet fully understood. A significant gap exists in comprehending the correlation between the CCl4-induced pulmonary fibrosis model in rats and pulmonary fibrosis in humans, particularly concerning pathogenesis and pathological characteristics. This study aims to elucidate the relevance of the CCl4-induced rat model in replicating the pathogenesis of human pulmonary fibrosis. By systematically comparing and analyzing the pathological changes, molecular regulatory networks, and cellular response characteristics of both models, this research endeavors to provide a robust theoretical foundation and a novel experimental framework for preclinical investigations into pulmonary fibrosis. Moreover, this study not only advances the understanding of the multi-organ toxicity mechanisms associated with CCl4 but also provides a crucial foundation in translational medicine for the development of novel therapeutic strategies targeting pulmonary fibrosis. Future research efforts will focus on elucidating the mechanisms of KA against HF through the integration of various omics approaches. Our study employed a rigorously designed animal model that accurately recapitulated the pathophysiological characteristics of liver fibrosis observed in humans. Through a blinded, randomized experimental protocol with an adequate sample size, we demonstrated a potent antifibrotic effect of kaempferol, thereby minimizing the risk of false-positive results. These findings underscore not only the therapeutic potential of kaempferol but also the translational value of our preclinical data for future clinical applications. This research establishes a robust theoretical foundation for subsequent clinical studies, following a clear translational pathway from mechanistic research to evidence generation, ultimately aiming at patient benefit. Although current investigations remain at the preclinical stage, utilizing both cellular and animal models, the observed strong efficacy and favorable safety profile of kaempferol suggest its significant promise as a potential clinical candidate for liver fibrosis treatment. These findings warrant further exploration through clinical trials to validate their therapeutic potential in human patients.

## 5 Conclusion

By integrating bibliometric and network pharmacology approaches, the study systematically screened and validated the anti-hepatic fibrosis mechanisms of the active component KA found in TCM. In a CCl_4_-induced rat model of HF, KA exhibited significant ameliorative effects on pathological conditions. Transcriptomic analysis indicated that KA facilitates the activation of the PPAR signaling pathway, reprogramming of fatty acid metabolism, regulation of retinol homeostasis, and maintenance of the dynamic balance within the arachidonic acid metabolic network by modulating core target genes such as *SCD1/SCD2*, *FADS2*, and *CYP4A8*. The research demonstrated that KA significantly suppresses the activation of HSCs and the accumulation of ECM by concurrently targeting the dysregulation of lipid metabolism and the inflammatory response pathways. This study provides a theoretical basis for the clinical use of KA in the treatment of liver fibrosis.

## Data Availability

The datasets presented in this study can be found in online repositories. The names of the repository/repositories and accession number(s) can be found below: https://www.ncbi.nlm.nih.gov/bioproject/, PRJNA992181.
