# Supplementary material for: Bibliometrics-guided cyberpharmacology and transcriptomics for multidimensional analysis of the antihepatic fibrosis mechanism of kaempferol
Source: Front Mol Biosci. 2025 Aug 29;12:1607103. doi: 10.3389/fmolb.2025.1607103 (PMC12426407; doi:10.3389/fmolb.2025.1607103)
Supplement: Supplementary file 1 [file Supplementaryfile1.docx]

1. *Codonopsis pilosula* Intersection targets with hepatic fibrosis

PGR NR3C2 PTGS2 RXRA SCN5A ADRB2 CHRM3 ADH1C NCOA1 PTGS1 ADRA2A SLC6A3 PLAU LTA4H CTRB1 CHRM1 ADRB1 ADRA1A CHRM2 ADRA1B GABRA1 NOS2 ESR1 AR ACHE SLC6A4 ESR2 MAPK14 GSK3B CHEK1 CCNA2 PKIA RELA EGFR AKT1 CCND1 BCL2L1 CDKN1A CASP9 MMP2 MMP9 MAPK1 IL10 RB1 TNF IL6 CASP3 TP53 NFKBIA XDH TOP1 MDM2 MMP1 PCNA ERBB2 PPARG HMOX1 ICAM1 MCL1 BIRC5 IL2 CCNB1 TYR IFNG IL4 TOP2A GSTP1 SLC2A4 INSR CD40LG PTGES MET OPRD1 TUBB1 NR3C1 MMP13 MMP8

1. *Prunus persica* Intersection targets with hepatic fibrosis

PGR PTGS2 GABRA1 ADH1C NR3C2 PTGS1 CHRM1 CHRM3 CHRM2 SLC6A3 ADRB2 SLC6A4 ADRA1B SCN5A RXRA KCNH2 CHRM4 ADRA1A BCL2 BAX CASP9 CASP3 CASP8 PRKCA PON1

1. *Bupleurum chinense* Intersection targets with hepatic fibrosis

PTGS1 PTGS2 RXRA PGR NR3C2 ADH1C NCOA1 ADRA2A SLC6A3 ADRB2 PLAU LTA4H CTRB1 CHRM3 CHRM1 ADRB1 SCN5A ADRA1A CHRM2 ADRA1B GABRA1 NOS2 ESR1 AR ESR2 MAPK14 GSK3B CCNA2 PYGM CHEK1 F7 ACHE RELA XDH NCF1 OLR1 IKBKB AKT1 BCL2 BAX TNF AHSA1 CASP3 MAPK8 MMP1 STAT1 PPARG HMOX1 CYP3A4 CYP1A2 CYP1A1 ICAM1 SELE VCAM1 NR1I2 CYP1B1 HAS2 GSTP1 AHR PSMD3 SLC2A4 NR1I3 INSR GSTM1 SLPI KCNH2 MMP3 EGFR CCND1 BCL2L1 CDKN1A EIF6 CASP9 MMP2 MMP9 MAPK1 IL10 EGF RB1 IL6 TP53 ELK1 NFKBIA POR ODC1 CASP8 TOP1 RAF1 PRKCA HIF1A ERBB2 ACACA CAV1 MYC F3 GJA1 IL1B CCL2 PTGER3 CXCL8 PRKCB BIRC5 DUOX2 HSPB1 IL2 CCNB1 PLAT THBD SERPINE1 IFNG IL1A MPO TOP2A NFE2L2 CXCL11 CXCL2 CLDN4 PPARA PPARD HSF1 CRP CXCL10 CHUK SPP1 RUNX2 RASSF1 E2F1 E2F2 ACP3 CTSD IGFBP3 IGF2 CD40LG IRF1 ERBB3 PON1 PCOLCE NPEPPS HK2 RASA1

1. *Astragalus membranaceus* Intersection targets with hepatic fibrosis

PGR NOS2 PTGS1 AR SCN5A PTGS2 ESR2 CHEK1 CHRM3 CHRM1 CHRM2 ADRA1B GABRA1 ADH1C RXRA ESR1 MAPK14 GSK3B CCNA2 PYGM NCOA1 F7 ACHE RELA XDH NCF1 OLR1 ADRB1 HTR3A ADRA2C ADRB2 KCNH2 CHRM4 OPRD1 ADRA1A SLC6A3 SLC6A4 RXRB KDR MET PKIA PPARG IL4 ATP5F1B MT-ND6 HSD3B1 IKBKB AKT1 BCL2 BAX TNF AHSA1 CASP3 MAPK8 MMP1 STAT1 HMOX1 CYP3A4 CYP1A2 CYP1A1 ICAM1 SELE VCAM1 NR1I2 CYP1B1 HAS2 GSTP1 AHR PSMD3 SLC2A4 NR1I3 INSR GSTM1 SLPI MMP3 EGFR CCND1 BCL2L1 CDKN1A EIF6 CASP9 PLAU MMP2 MMP9 MAPK1 IL10 EGF RB1 IL6 TP53 ELK1 NFKBIA POR ODC1 CASP8 TOP1 RAF1 PRKCA HIF1A ERBB2 ACACA CAV1 MYC F3 GJA1 IL1B CCL2 PTGER3 CXCL8 PRKCB BIRC5 DUOX2 HSPB1 IL2 CCNB1 PLAT THBD SERPINE1 IFNG IL1A MPO TOP2A NFE2L2 CXCL11 CXCL2 CLDN4 PPARA PPARD HSF1 CRP CXCL10 CHUK SPP1 RUNX2 RASSF1 E2F1 E2F2 ACP3 CTSD IGFBP3 IGF2 CD40LG IRF1 ERBB3 PON1 PCOLCE NPEPPS HK2 RASA1

1. *Glycyrrhiza uralensis* Intersection targets with hepatic fibrosis

PTGS1 CHRM3 SCN5A PTGS2 HTR3A RXRA ADRA1B CHRM1 ADRB2 ESR1 SLC6A4 PKIA PGR NOS2 KDR MAPK14 GSK3B CHEK1 CCNA2 AR ESR2 CHRM4 ADRA1A CHRM2 SLC6A3 MAPK10 OPRD1 PYGM NCOA1 F7 ACHE GABRA1 RELA XDH NCF1 OLR1 NR3C2 ADRB1 LTA4H PPARG IL4 ATP5F1B MT-ND6 HSD3B1 IKBKB AKT1 BCL2 BAX TNF AHSA1 CASP3 MAPK8 MMP1 STAT1 HMOX1 CYP3A4 CYP1A2 CYP1A1 ICAM1 SELE VCAM1 NR1I2 CYP1B1 HAS2 GSTP1 AHR PSMD3 SLC2A4 NR1I3 INSR GSTM1 SLPI MAPK3 MAPK1 FASN LDLR CAT MTTP APOB PLB1 HMGCR PPARA SREBF1 GSR ABCC1 ADIPOQ SOAT2 GOT1 SOAT1 KCNH2 RXRB STAT3 CCND1 EIF6 RB1 FOSL2 MMP3 EGFR BCL2L1 CDKN1A CASP9 PLAU MMP2 MMP9 IL10 EGF IL6 TP53 ELK1 NFKBIA POR ODC1 CASP8 TOP1 RAF1 PRKCA HIF1A ERBB2 ACACA CAV1 MYC F3 GJA1 IL1B CCL2 PTGER3 CXCL8 PRKCB BIRC5 DUOX2 HSPB1 IL2 CCNB1 PLAT THBD SERPINE1 IFNG IL1A MPO TOP2A NFE2L2 CXCL11 CXCL2 CLDN4 PPARD HSF1 CRP CXCL10 CHUK SPP1 RUNX2 RASSF1 E2F1 E2F2 ACP3 CTSD IGFBP3 IGF2 CD40LG IRF1 ERBB3 PON1 PCOLCE NPEPPS HK2 RASA1
